# Supplementary material for: Examination of acute spin exercise on GABA levels in aging and stroke: The EASE study protocol
Source: PLoS One. 2024 Jul 15;19(7):e0297841. doi: 10.1371/journal.pone.0297841 (PMC11249249; doi:10.1371/journal.pone.0297841)
Supplement: S2 File — (DOCX) [file pone.0297841.s002.docx]

| **1. Hardware** | |
| --- | --- |
| a. Field strength [T] | 3 T |
| b. Manufacturer | Siemens |
| c. Model (software version if available) | Prisma (Syngo VE11C) |
| d. RF coils: nuclei (transmit/receive), number of channels, type, body part | 32-channel head coil |
| **2. Acquisition** | |
| a. Pulse sequence | MEGA-PRESS |
| b. Volume of interest (VOI) locations | Dominant (left) primary motor cortex |
| c. Nominal VOI size [cm^3^, mm^3^] | 3 × 3 × 3 cm^3^ = 27mL |
| d. Repetition time (*T*_R_), echo time (*T*_E_) [ms, s] | *T*_R_ 2000 ms, *T_E_* 68 *ms* |
| e. Total number of excitations or acquisitions per spectrum | 174 averages (87 pairs) |
| f. Additional sequence parameters (spectral width in Hz, number of spectral points, frequency offsets) | Spectral BW=4000 Hz, Num Spectral points=4128, edit ON=1.9ppm, edit OFF=7.5 ppm, Reference=3 ppm |
| g. Water suppression method | VAPOR |
| h. Shimming method, reference peak, and thresholds | Field mapping with FAST(EST) MAP, followed by manual shimming of water to <19 Hz |
|  |  |

| **3. Data analysis methods and outputs** | |
| --- | --- |
| a. Analysis software | Gannett 3.3.1 |
| b. Output measures | GABA ratio to A) water reference and B) creatine; GABA levels across MRS runs will be normalized to first resting GABA acquisition in each session; GABA session dynamic range will be calculated as maximum-minimum GABA across within-session runs |
| c. Quantification references and assumptions, fitting model assumptions | Robust spectral registration; global zero-order phase correction to all transients by fitting 3 ppm Cr and 3.2 ppm Cho peaks; weighted averaging |
