# Supplementary material for: Examination of acute spin exercise on GABA levels in aging and stroke: The EASE study protocol
Source: PLoS One. 2024 Jul 15;19(7):e0297841. doi: 10.1371/journal.pone.0297841 (PMC11249249; doi:10.1371/journal.pone.0297841)
Supplement: S3 File — (PDF) [file pone.0297841.s003.pdf]

SOP BLOOD DRAW AT MAGNET

Lactate measurement

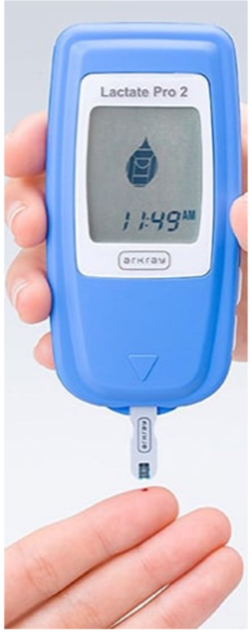

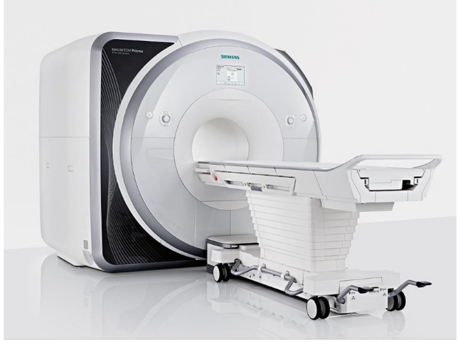

REQUIRED ITEMS

- Lactate monitor
- 2x2 Gauze - pack
- Lactate Test Strips – minimum 15 per person
- Alcohol swabs – pack
- Drummond 5μL transfer pipettes
- Hand towels
- Chucks
- Recording Sheet
- Lancet device and lancet
- Bottle of Water for participant
- Spray bottle of water for chest strap HR monitor (dampen)

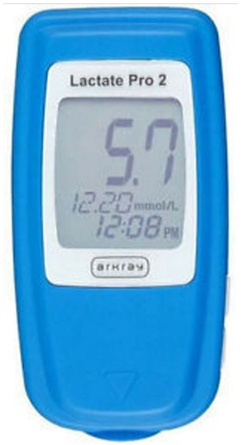

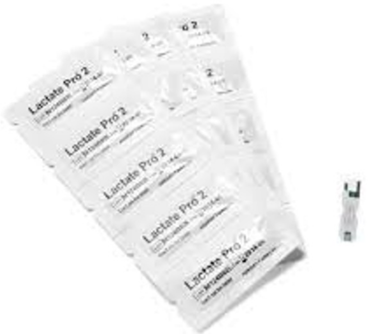

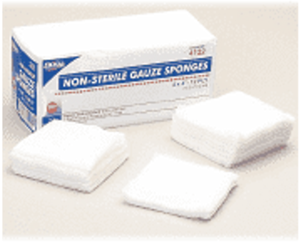

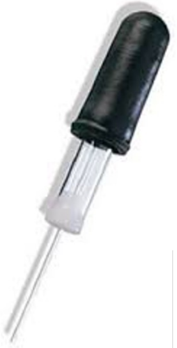

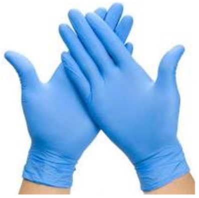

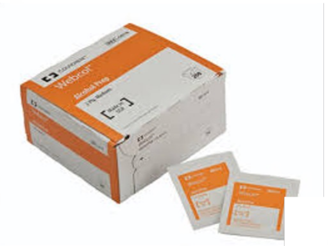

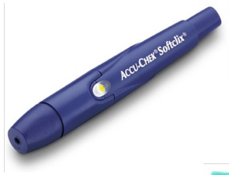

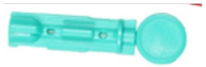

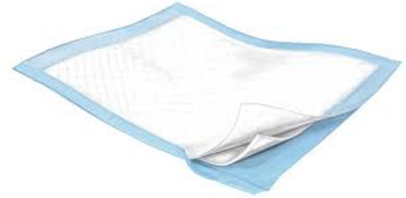

*Items not to scale
**Batteries sold separately

FINGER STICK FOR LACTATE OUTSIDE OF MAGNET

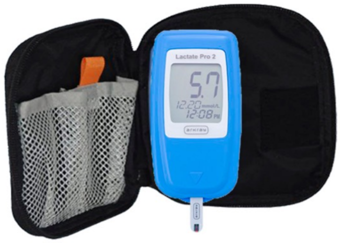

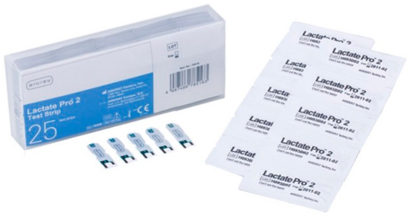

1) Use lateral portion of finger (ideally middle or ring finger) – index if otl[illegible] accessible
2) Clean area with alcohol swab
3) Let air dry (seconds)
4) Perform puncture with lancet (set to 5 for depth)
5) Allow first blood droplet of blood to form and then WIPE away with 2x2 gauze pad
6) ONLY use second droplet to sample lactate
7) Insert lactate test strip to lactate monitor (it will turn on monitor)
8) Place end of lactate strip on second drop of blood (less than 5 seconds should elapse between puncture and sampling)

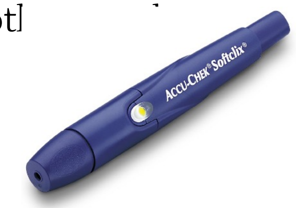

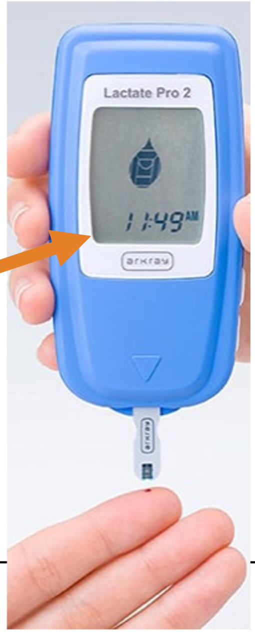

MRI IMPORTANT NOTES:

- Hand must be kept warm when in the magnet (use DRY towel)
- Spring in the Lancet holder is Magnetic (magnet will grab it – do **not** let it go)
    - (We will glue a holder string onto the lancet holder)
- To draw blood in magnet following must be prepped:

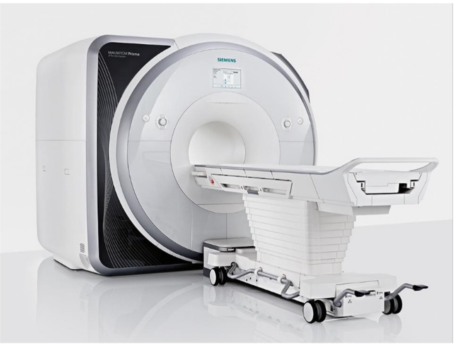

1. Clean 4/5µL pipette needs to be fitted to bulb cylinder
2. Prepare 8 or 10 2x2 gauze pads
3. Remove alcohol swab from packet and place on top of gauze pads
4. Bring paper towel or hand towel to hold material when going into magnet room

---

- **NOTE: This needs practice. If there is an error, this timepoint measure is missed.**

FINGER STICK FOR LACTATE INSIDE MAGNET

1) Use same finger as outside magnet
2) Remove hand towel and clean finger stick area with alcohol swab
3) Let air dry (5 or 6 seconds) then use clean gauze to wipe
4) Perform puncture with lancet (set to 5 for depth)
5) Allow first blood droplet of blood to form and then WIPE away with 2x2 gauze pad
6) ONLY use second droplet to sample lactate
7) Remove blood with pipette (capillary action will draw up blood)
8) Place gauze on finger and press participants fingers together to indicate they should hold the gauze for clotting
9) Cover hand with hand towel for warmth

- **NOTE: This needs practice. If there is an error, this timepoint measure is missed.**

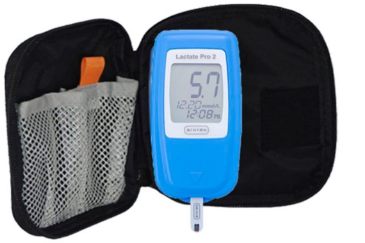
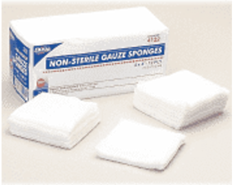

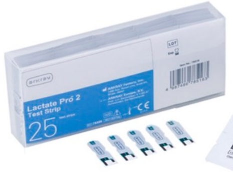

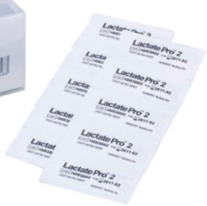
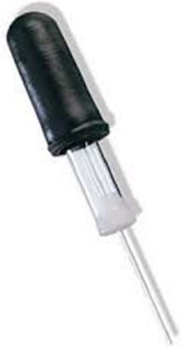
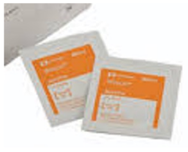
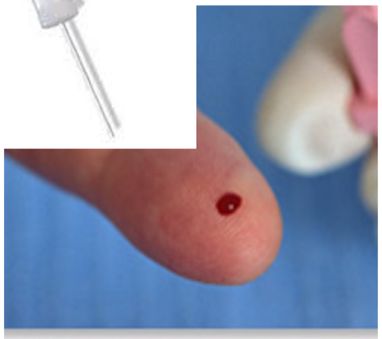

HOW MUCH BLOOD?

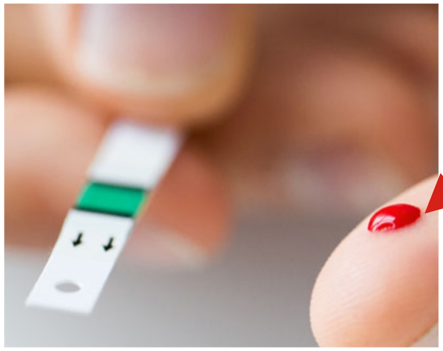

This is too much

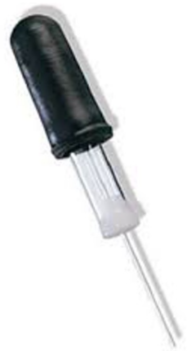

This is too little

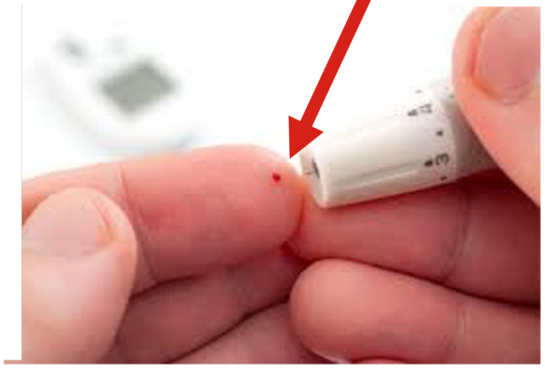

JUST RIGHT AMOUNT

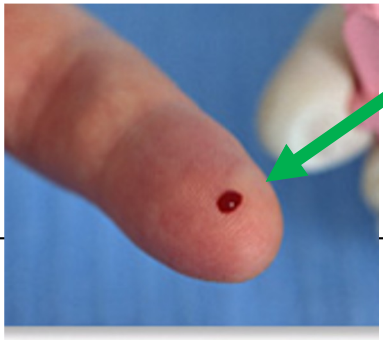

MEASURE LACTATE - MRI

- PREP:
- Open a lactate strip but do not remove from packet
- Place an unused lactate strip on the chuck (do not open)
- Wipe off with alcohol and then gauze to clean (ensure it is dry)
- Prior to going into magnet (remove lactate strip from opened packet and place in Lactate Pro
- Retrieve sample from magnet using above procedure
- MEASURE:
- Put finger on back of dropper squeeze blood drop onto lactate strip (bubble may form, it’s ok)
- Dip Lactate Pro active strip into blood sample

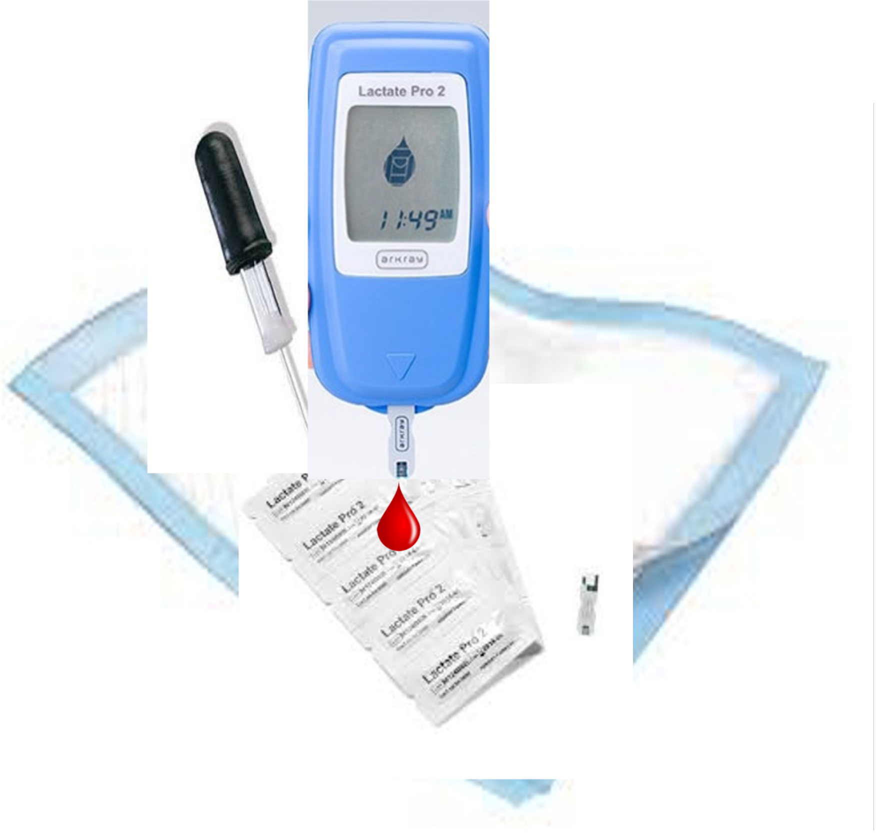

- **NOTE: This needs practice. If there is an error, this timepoint measure is missed.**

RECORD EVERYTHING

Lactate			
Time	Measurement	Value (mmol/L)	QC
	Rest Lactate1		
	Rest Lactate2		
	MR_Lactate_1		
	Lactate_spin1		
	Lactate_spin2		
	Lactate_spin3		
	Lactate_spin4		
	Lactate_spin5		
	MR_Lactate_2		
	MR_Lactate_3		
	MR_Lactate_4		
	MR_Lactate_5		

Ensure you have XLS sheet that has data recording
