# Supplementary material for: Examination of acute spin exercise on GABA levels in aging and stroke: The EASE study protocol
Source: PLoS One. 2024 Jul 15;19(7):e0297841. doi: 10.1371/journal.pone.0297841 (PMC11249249; doi:10.1371/journal.pone.0297841)
Supplement: S4 File — (DOCX) [file pone.0297841.s004.docx]

STROBE Statement—checklist of items that should be included in reports of observational studies

|  | Item No. | Recommendation | Page  No. | Relevant text from manuscript |
| --- | --- | --- | --- | --- |
| **Title and abstract** | 1 | (*a*) Indicate the study’s design with a commonly used term in the title or the abstract | 1 | Examination of Acute Spin Exercise on GABA Levels in Aging and Stroke: The EASE Study Protocol |
|  |  | (*b*) Provide in the abstract an informative and balanced summary of what was done and what was found | 2 | Background: Rehabilitating upper extremity function in persons with chronic stroke remains challenging because of considerable variation in response to treatment. |
| Introduction | | | |  |
| Background/rationale | 2 | Explain the scientific background and rationale for the investigation being reported | 4,5,6 | Rationale and existing literature are stated in the introduction section |
| Objectives | 3 | State specific objectives, including any prespecified hypotheses | 6 | Objectives are listed on page 6 last paragraph of Introduction |
| Methods | | | |  |
| Study design | 4 | Present key elements of study design early in the paper | 6 | First paragraph of Methods. |
| Setting | 5 | Describe the setting, locations, and relevant dates, including periods of recruitment, exposure, follow-up, and data collection | 7,8 | Listed in Participants section |
| Participants | 6 | (*a*) *Cohort study*—Give the eligibility criteria, and the sources and methods of selection of participants. Describe methods of follow-up  *Case-control study*—Give the eligibility criteria, and the sources and methods of case ascertainment and control selection. Give the rationale for the choice of cases and controls  *Cross-sectional study*—Give the eligibility criteria, and the sources and methods of selection of participants | 4 | Established on page 4 as cross-sectional study. |
|  |  | (*b*) *Cohort study*—For matched studies, give matching criteria and number of exposed and unexposed  *Case-control study*—For matched studies, give matching criteria and the number of controls per case |  |  |
| Variables | 7 | Clearly define all outcomes, exposures, predictors, potential confounders, and effect modifiers. Give diagnostic criteria, if applicable | 7,8,9,21, 22-23 | Main outcome measures are listed in Table 2, Figure 1 and text: pgs 7-21. Assessed cognitive/psychological variables are listed in Table 1. |
| Data sources/ measurement | 8* | For each variable of interest, give sources of data and details of methods of assessment (measurement). Describe comparability of assessment methods if there is more than one group | 7,8,9,21, 22-23 | Outcome measures are listed in Table 2, Figure 1 and text: pgs 7-16. |
| Bias | 9 | Describe any efforts to address potential sources of bias | Table 1 | We attempted to reduce bias by excluding contraindicated participants in Table 1. Our analysis was adjusted on type on context to reduce bias expected. |
| Study size | 10 | Explain how the study size was arrived at | 21 | Listed in Power analysis and Sample size section. |

Continued on next page

| Quantitative variables | 11 | Explain how quantitative variables were handled in the analyses. If applicable, describe which groupings were chosen and why | 22-23 | Listed in Methods: Analysis section on pages 22-23. |
| --- | --- | --- | --- | --- |
| Statistical methods | 12 | (*a*) Describe all statistical methods, including those used to control for confounding | 22 | Listed in Methods: Analysis section on page 22 and 23 |
|  |  | (*b*) Describe any methods used to examine subgroups and interactions | 22 | Listed in Methods: Analysis section on page 22 and 23 |
|  |  | (*c*) Explain how missing data were addressed | N/A | Only participants with all sessions of data will be analyzed. We budgeted for dropout of 20% in the study to account for missing data (see participants section) |
|  |  | (*d*) *Cohort study*—If applicable, explain how loss to follow-up was addressed  *Case-control study*—If applicable, explain how matching of cases and controls was addressed  *Cross-sectional study*—If applicable, describe analytical methods taking account of sampling strategy | Cross-sectional listed on page 7-10 | Cross-sectional study for three groups will be employed using t-tests and regression. Sampling bias is limited by participant inclusion criteria. |
|  |  | (*e*) Describe any sensitivity analyses | 21 | Analogous report is listed in Power Analysis and Sample Size section from previous studies. |
| Results | | | | |
| Participants | 13* | (a) Report numbers of individuals at each stage of study—eg numbers potentially eligible, examined for eligibility, confirmed eligible, included in the study, completing follow-up, and analysed | N/A | Dropout is accounted for in inclusion criteria and participants with missing sessions will be excluded from analysis. |
|  |  | (b) Give reasons for non-participation at each stage | N/A | All participants are informed of requirement for attendance of all sessions during consenting process. Dropout was accounted for in inclusion criteria and participants with missing sessions will be excluded from analysis. |
|  |  | (c) Consider use of a flow diagram | Table 2/Figure 1 | See Table 2 and Figure 1 for study session flow. |
| Descriptive data | 14* | (a) Give characteristics of study participants (eg demographic, clinical, social) and information on exposures and potential confounders | Table 1 | See Table 1 for inclusion criteria |
|  |  | (b) Indicate number of participants with missing data for each variable of interest | N/A | All participants are informed of requirement for attendance of all sessions during consenting process. Dropout was accounted for in inclusion criteria and participants with missing sessions will be excluded from analysis. |
|  |  | (c) *Cohort study*—Summarise follow-up time (eg, average and total amount) | N/A |  |
| Outcome data | 15* | *Cohort study*—Report numbers of outcome events or summary measures over time | *N/A* |  |
|  |  | *Case-control study—*Report numbers in each exposure category, or summary measures of exposure | *N/A* |  |
|  |  | *Cross-sectional study—*Report numbers of outcome events or summary measures | 7,8,9,21, 22-23 | Outcome measures are listed in Table 2, Figure 1 and text: pgs 7-16. |
| Main results | 16 | (*a*) Give unadjusted estimates and, if applicable, confounder-adjusted estimates and their precision (eg, 95% confidence interval). Make clear which confounders were adjusted for and why they were included | 22-23 | Power analysis lists unadjusted estimates. Confounders limited by selection criteria. |
|  |  | (*b*) Report category boundaries when continuous variables were categorized | N/A |  |
|  |  | (*c*) If relevant, consider translating estimates of relative risk into absolute risk for a meaningful time period | N/R | N/R |

Continued on next page

| Other analyses | 17 | Report other analyses done—eg analyses of subgroups and interactions, and sensitivity analyses | N/A |  |
| --- | --- | --- | --- | --- |
| Discussion | | | | |
| Key results | 18 | Summarise key results with reference to study objectives | 23-24 | Listed in Discussion (first three paragraphs) |
| Limitations | 19 | Discuss limitations of the study, taking into account sources of potential bias or imprecision. Discuss both direction and magnitude of any potential bias | 24 | Listed in Discussion -see lines 21-23 |
| Interpretation | 20 | Give a cautious overall interpretation of results considering objectives, limitations, multiplicity of analyses, results from similar studies, and other relevant evidence | 24 | Limitations listed in discussion -see line 21-23 |
| Generalisability | 21 | Discuss the generalisability (external validity) of the study results | 24 | Generalization limitations listed in discussion -see lines 21-23 |
| Other information | |  | | |
| Funding | 22 | Give the source of funding and the role of the funders for the present study and, if applicable, for the original study on which the present article is based | 25 | Funding information listed on page 25. |

*Give information separately for cases and controls in case-control studies and, if applicable, for exposed and unexposed groups in cohort and cross-sectional studies.

**Note:** An Explanation and Elaboration article discusses each checklist item and gives methodological background and published examples of transparent reporting. The STROBE checklist is best used in conjunction with this article (freely available on the Web sites of PLoS Medicine at http://www.plosmedicine.org/, Annals of Internal Medicine at http://www.annals.org/, and Epidemiology at http://www.epidem.com/). Information on the STROBE Initiative is available at www.strobe-statement.org.
