# Supplementary material for: Examination of acute spin exercise on GABA levels in aging and stroke: The EASE study protocol
Source: PLoS One. 2024 Jul 15;19(7):e0297841. doi: 10.1371/journal.pone.0297841 (PMC11249249; doi:10.1371/journal.pone.0297841)
Supplement: S1 File — (PDF) [file pone.0297841.s001.pdf]

MRI Scan/Data Sheet- EASE

Subject ID#:____________________ Experimenters:______________________________ Date:_________________________

Session:_________________________ Technologist:________________________________ Arrival Time:___________________

Scanning				Physiological			Lactate			
#	Scan	Start Time	QC	Time	HR	RPE (6-20)	Time	Measurement	Value (mmol/L)	QC
1	Localizer									
2	T1 MPRAGE									
3	Fastestmap_M1									
4	Manual Shim									
5	PRESSTE20_H20_Pre									
6	MPRESS_GABACalibration									
7	MPRESS_REST1_GABA_pre									
8	MPRESS_ENT1_GABA_pre									
9	MPRESS_ENT2_GABA_pre									
10	MPRESS_REC1_GABA_pre									
11	MPRESS_REST2_GABA_pre									
