# Supplementary material for: Examination of acute spin exercise on GABA levels in aging and stroke: The EASE study protocol
Source: PLoS One. 2024 Jul 15;19(7):e0297841. doi: 10.1371/journal.pone.0297841 (PMC11249249; doi:10.1371/journal.pone.0297841)
Supplement: Attachment — Submitted filename: Response_to_Review.docx [file pone.0297841.s005.docx]

PONE-D-23-15402

Examination of Acute Spin Exercise on GABA Levels in Aging and Stroke: The EASE Study Protocol

PLOS ONE

Dear Dr. McGregor,

Thank you for submitting your manuscript to PLOS ONE. After careful consideration, we feel that it has merit but does not fully meet PLOS ONE’s publication criteria as it currently stands. Therefore, we invite you to submit a revised version of the manuscript that addresses the points raised during the review process.

Please submit your revised manuscript by Sep 25 2023 11:59PM. If you will need more time than this to complete your revisions, please reply to this message or contact the journal office at plosone@plos.org. When you're ready to submit your revision, log on to https://www.editorialmanager.com/pone/ and select the 'Submissions Needing Revision' folder to locate your manuscript file.

Please include the following items when submitting your revised manuscript:

A rebuttal letter that responds to each point raised by the academic editor and reviewer(s). You should upload this letter as a separate file labeled 'Response to Reviewers'.

A marked-up copy of your manuscript that highlights changes made to the original version. You should upload this as a separate file labeled 'Revised Manuscript with Track Changes'.

An unmarked version of your revised paper without tracked changes. You should upload this as a separate file labeled 'Manuscript'.

If you would like to make changes to your financial disclosure, please include your updated statement in your cover letter. Guidelines for resubmitting your figure files are available below the reviewer comments at the end of this letter.

If applicable, we recommend that you deposit your laboratory protocols in protocols.io to enhance the reproducibility of your results. Protocols.io assigns your protocol its own identifier (DOI) so that it can be cited independently in the future. For instructions see: https://journals.plos.org/plosone/s/submission-guidelines#loc-laboratory-protocols. Additionally, PLOS ONE offers an option for publishing peer-reviewed Lab Protocol articles, which describe protocols hosted on protocols.io. Read more information on sharing protocols at https://plos.org/protocols?utm_medium=editorial-email&utm_source=authorletters&utm_campaign=protocols.

We look forward to receiving your revised manuscript.

Kind regards,

Nadinne Alexandra Roman, Ph.D.

Academic Editor

PLOS ONE

Journal Requirements:

When submitting your revision, we need you to address these additional requirements.

1. Please ensure that your manuscript meets PLOS ONE's style requirements, including those for file naming. The PLOS ONE style templates can be found at

https://journals.plos.org/plosone/s/file?id=wjVg/PLOSOne_formatting_sample_main_body.pdf and

https://journals.plos.org/plosone/s/file?id=ba62/PLOSOne_formatting_sample_title_authors_affiliations.pdf

2. Please provide additional details regarding participant consent. In the ethics statement in the Methods and online submission information, please ensure that you have specified (1) whether consent was informed and (2) what type you obtained (for instance, written or verbal, and if verbal, how it was documented and witnessed). If your study included minors, state whether you obtained consent from parents or guardians. If the need for consent was waived by the ethics committee, please include this information.

If you are reporting a retrospective study of medical records or archived samples, please ensure that you have discussed whether all data were fully anonymized before you accessed them and/or whether the IRB or ethics committee waived the requirement for informed consent. If patients provided informed written consent to have data from their medical records used in research, please include this information.

3. In your Data Availability statement, you have not specified where the minimal data set underlying the results described in your manuscript can be found. PLOS defines a study's minimal data set as the underlying data used to reach the conclusions drawn in the manuscript and any additional data required to replicate the reported study findings in their entirety. All PLOS journals require that the minimal data set be made fully available. For more information about our data policy, please see http://journals.plos.org/plosone/s/data-availability.

Upon re-submitting your revised manuscript, please upload your study’s minimal underlying data set as either Supporting Information files or to a stable, public repository and include the relevant URLs, DOIs, or accession numbers within your revised cover letter. For a list of acceptable repositories, please see http://journals.plos.org/plosone/s/data-availability#loc-recommended-repositories. Any potentially identifying patient information must be fully anonymized.

Important: If there are ethical or legal restrictions to sharing your data publicly, please explain these restrictions in detail. Please see our guidelines for more information on what we consider unacceptable restrictions to publicly sharing data: http://journals.plos.org/plosone/s/data-availability#loc-unacceptable-data-access-restrictions. Note that it is not acceptable for the authors to be the sole named individuals responsible for ensuring data access.

We will update your Data Availability statement to reflect the information you provide in your cover letter.

4. Please include captions for your Supporting Information files at the end of your manuscript, and update any in-text citations to match accordingly. Please see our Supporting Information guidelines for more information: http://journals.plos.org/plosone/s/supporting-information.

[Note: HTML markup is below. Please do not edit.]

Reviewers' comments:

Reviewer's Responses to Questions

Comments to the Author

1. Does the manuscript provide a valid rationale for the proposed study, with clearly identified and justified research questions?

The research question outlined is expected to address a valid academic problem or topic and contribute to the base of knowledge in the field.

Reviewer #1: Yes

Reviewer #2: Yes

Reviewer #3: Yes

2. Is the protocol technically sound and planned in a manner that will lead to a meaningful outcome and allow testing the stated hypotheses?

The manuscript should describe the methods in sufficient detail to prevent undisclosed flexibility in the experimental procedure or analysis pipeline, including sufficient outcome-neutral conditions (e.g. necessary controls, absence of floor or ceiling effects) to test the proposed hypotheses and a statistical power analysis where applicable. As there may be aspects of the methodology and analysis which can only be refined once the work is undertaken, authors should outline potential assumptions and explicitly describe what aspects of the proposed analyses, if any, are exploratory.

Reviewer #1: Yes

Reviewer #2: Yes

Reviewer #3: Partly

3. Is the methodology feasible and described in sufficient detail to allow the work to be replicable?

Descriptions of methods and materials in the protocol should be reported in sufficient detail for another researcher to reproduce all experiments and analyses. The protocol should describe the appropriate controls, sample size calculations, and replication needed to ensure that the data are robust and reproducible.

Reviewer #1: Yes

Reviewer #2: Yes

Reviewer #3: No

We have attempted to improve the clarity of the methods and materials section to better promote reproduction of the protocol and subsequent data.

4. Have the authors described where all data underlying the findings will be made available when the study is complete?

The PLOS Data policy requires authors to make all data underlying the findings described in their manuscript fully available without restriction, with rare exception, at the time of publication. The data should be provided as part of the manuscript or its supporting information, or deposited to a public repository. For example, in addition to summary statistics, the data points behind means, medians and variance measures should be available. If there are restrictions on publicly sharing data—e.g. participant privacy or use of data from a third party—those must be specified.

Reviewer #1: Yes

Reviewer #2: No

The Department of Veterans Affairs owns the resultant original data from the project. We will make de-identified copies of the data available on the website osf.io in accordance with the publishing recommendation in the data access protocol of the project. We have clarified this in the Dissemination Policy.

Reviewer #3: Yes

5. Is the manuscript presented in an intelligible fashion and written in standard English?

PLOS ONE does not copyedit accepted manuscripts, so the language in submitted articles must be clear, correct, and unambiguous. Any typographical or grammatical errors should be corrected at revision, so please note any specific errors here.

Reviewer #1: Yes

Reviewer #2: Yes

Reviewer #3: Yes

6. Review Comments to the Author

Please use the space provided to explain your answers to the questions above and, if applicable, provide comments about issues authors must address before this protocol can be accepted for publication. You may also include additional comments for the author, including concerns about research or publication ethics.

You may also provide optional suggestions and comments to authors that they might find helpful in planning their study.

(Please upload your review as an attachment if it exceeds 20,000 characters)

Reviewer #1: This manuscript consisted of valuable study protocol for investigation after stroke and elder subjects.

Reviewer #2: I have no comments. The proposed study is feasible and sound.

However, the authors have failed to provide appropriate IRB approval from the Atlanta VAMC and Emory University Hospital, which contradicts PLOS ONE's submission rules for Study Protocols.

We thank the reviewer for this statement and have added the necessary language for ethics board approval. A copy of the IRB Approval letter has been added to the submission, as well. PAGE 4

Reviewer #3: Thank you for the opportunity to review the protocol ‘Effects of acute exercise on functional GABA levels’. It is an interesting study and valuable to the field, investigating differences in GABA levels across healthy aging and in stroke and how these change with exercise. I have some concerns about the write up of the protocol, as well as a few methodological comments. I appreciate data collection has already commenced and these may not be able to be implemented. My knowledge on exercise assessments is limited, so I apologise for any naivete in questions around the exercise portion of the protocol.

Major concerns:

- Clarity of aims and links to analysis. My understanding is there are two key aims: 1) the change in GABA levels following exercise within each group, and if they differ between groups and 2) how GABA levels relate to motor acquisition. 2 is divided into multiple parts: for healthy controls, the motor learning is being assessed within the scanner and simultaneously measuring changes in GABA levels using functional MRS, while for stroke patients resting GABA levels are being linked to out of scanner motor learning. This needs to be clearer throughout the manuscript.

We appreciate the comment and have clarified the methods with respect to the motor learning comparators. See Outcomes, Page 16.

Introduction: the final paragraph jumps around in regards to questions and hypotheses.

We have revised the introduction’s hypothesis summary section to clarify the specific hypotheses in the project. We have added the project’s Specific Aims and associated hypotheses to the text on page 4.

Methods: The first two paragraphs, beginning page 6, line 22, jumps around a lot and does not clearly link your aims to your design

The reviewer’s comment on adding the specific hypotheses to the project’s Aims was very helpful. This helped the flow of the first component of the Methods section. We have added the project specific aims and hypotheses, which better supports the analysis section.

Primary outcome on page 11 lists change in metabolites levels as primary outcome: this should just be GABA levels. This also is contradicted in the first paragraph on page 22, which lists MRS measures as secondary outcomes. Additionally in this paragraph it describes MRI as an outcome, which is not mentioned elsewhere.

We appreciate the comment and have made the suggested changes to the text's outcome measures.

- MRS acquisition and analysis

Much more description is needed in regards to the acquisition parameters and clarification in the analysis is required.

Acquisition: how is the voxel placed on the sensorimotor cortex? What anatomical guidelines are used? How are you ensuring replicability/overlap as mentioned on page 18, lines 12-14. More detail is needed in regards to the MRI/MRS procedure (see Lin et al, 2021, NMR Biomed for reporting of MRS parameters) and how it differs between healthy controls and participants with stroke: it appears the healthy control MRI session must be much longer than the healthy controls.

We thank the reviewer for advising us of this. We have added more detail regarding the acquisition parameters including the checklist as recommended by Lin et al., (2021) as an appendix. We have also added more detail of the voxel placement within and across acquisition sessions. Pages 11 and 12. We used anatomical guidelines as outlined by Yousry et al., 1997. I believe the reviewer means the healthy control MRI session was longer than the stroke session. If so, this is correct, the sessions with participants with stroke did not involve motor learning due to the individual differences in fractionated finger movements.

I have a concern regarding motion across the session, especially given the lactate sampling throughout the MRS acquisition. At minimum, I suggest repeating a brief localiser or other anatomical scan to assess for bulk motion changes throughout the session for scans that have not yet been conducted. Examination of the MRS spectra for any suggestion of motion should also be carefully completed.

Our apologies. This information was omitted from the previous version of the manuscript. We have added more detail about how we attempted to limit head motion. Indeed, the lactate acquisition was a concern for our group, so we generated a how-to manual for study staff to ensure repeatability and limit displacement of the participant. We acquired another localizer image during piloting for reference and monitored the GABA spectra for changes at each sampling interval. Considerations included providing clear instruction with positional reference and warming the finger with a heated facecloth to limit vascular constriction. We have also added more detail regarding the analysis parameters implemented in Gannett 3.3.2.

Analysis: how will preprocessing be conducted? ‘correct eddy currents, motion correction, retrospective removal of frequency and phase drifts, spectral alignment, and nuisance peak removal’? Within Gannet? The ‘we will extract GABA+ levels in gray and white matter against measures of water reference’? Are you referring to the alpha-correction as per Harris et al, 2015, J Magn Reson Imaging? Please clarify. How will normalization to rest GABA+ levels be done? How is GABA+ dynamic range defined or calculated? The analysis regarding the effects of exercise on GABA+ levels is unclear to me, as is the link for motor learning. Please provide more detail.

We have added much more detail in the text in regard to the data processing and timing considerations for the acquisition (see also new Appendix: MRS_Parameters Table).

We largely employed the default processing steps within Gannett 3.3.2, which included:

- Eddy correction (from Mikkelson et al., 2020)
- Spectral registration (from Near et al., 2015)
- Water referenced tissue-correction (from Harris et al., 2015)

In addition, we have added appropriate references in the text for definitions of normalization and dynamic range.

Will the paired-t-test be conducted for both regions for the participants with stroke? I also recommend considering including a secondary analysis using creatine referenced values to investigate any potential water signal changes and processing NAA levels or some other control metabolite to ensure findings are specific to GABA levels. I recommend keeping tracking of time from exercise until MRS scans, especially as it may take longer to set up the older participants again in the scanner and investigating any potential effects of time delay.

For GABA levels at rest in the dominant motor cortex, we will employ a linear mixed model analysis repeated measures analysis of GABA spectra with groups (younger, older, stroke) as between and session (pre/post) while holding subjects as random. A second-level analyses will be completed to compare dominant and non-dominant hemispheres in participants with stroke. The suggestion of a control metabolite (Cr) is well-met and we have added language to describe this in the text.

The comment about time recording is very astute. We implemented a robust time-keeping regimen to record the start time of all measurements (exercise [heart rate/lactate], imaging [runs/spectral evaluation/lactate draws], and session transitions. We have provided a sample acquisition sheet for these measures for reference.

- Exercise test: do participants do both the maximal and submaximal test? Are the outcomes from the exercise tests used? I think a little more detail on the description of speed and incline and how this test administered would be appropriate, or a citation of the methods used.

Our apologies, we have clarified this in the text. The submaximal exercise test was not performed in this project, and was a contingency concern if participants were contraindicated for maximal testing by a physician. This should have been removed. The exercise in the study refers to two activities: 1) VO2 assessment: the cardiorespiratory evaluation is done on a treadmill in session 1; 2) High intensity interval training (HIIT) cycling is done for the acute exercise in the imaging session.

- Power analysis: the power analysis described only seems to detail the within-subject effects of exercise. This needs more detail. Also please describe how you arrived at sample size for different groups, or that it is exploratory pilot.

This is a fair critique. The study was powered specifically for within-subjects effects. Group comparisons (younger, older and participants with stroke) for this study was exploratory.

- The analysis for outcomes from sessions 3 for stroke patients is not described.

Our apologies. We have addressed this oversight.

- The lactate analysis is not described and the sampling details are unclear. Will lactate be sampled between every MRS acquisition? It may be valuable to include it in the session flow schematic.

We have clarified the sampling details for the lactate acquisition. Yes, we will sample lactate between every scan. We added an appendix with information about the procedures in detail. In addition, the study flow is characterized in the project score sheet.

- Discussion: the discussion needs restructured, I suggest opening with a clear summary of the questions that will be asked, dedicated paragraphs to expected findings and then a clear summary. There are assertions of expected results that are not presently justified by analysis plan: ‘GABA concentration changes characterize flexibility in the GABA system during task engagement’ and ‘opportunity to evaluate the potential mechanistic role of GABA with respect to functional changes associated with motor skill performance’.

We have modified the text in the Discussion to provide a clearer summary of expected findings with respect to the project’s hypotheses.

Medium concerns:

- The sedentary individuals is not motivated in the introduction and is only introduced in the eligibility table (table 1).

We have clarified this in the text.

- It may be valuable to split out Figure 1 into more figures or at least make it larger

We have enlarged the image and will upload a scaled version to the site.

- I am unsure if it would be valuable to consider the learning timeframe outside the scanner (the pre-training). Would practice effects come in to this?

We have clarified this in the text. We limited the maximum familiarization time to 5 minutes of task execution.

- Is the alpha level stated on page 22, line 17, the one that will be Bonferroni adjusted for FWE?

I believe the reviewer may have meant the text under Behavioral Task Analysis (last sentence). We have clarified that we meant uncorrected alpha.

- Would like a little bit of information about how much the healthy controls are told about what they are expected to learn? What if participants do not learn the sequence?

We have added the text instructions into the Methods for clarity.

Small concerns:

- Throughout the manuscript, the tense switches between past and future, this should be made consistent

We have corrected tense shifts, as appropriate.

- Abstract, page 2:, line 9, should it be ‘limited BY neural damage, rather than to?

Yes, thank you for that correction.

- The two aims are not really addressed in the intro of the abstract, you could decrease the background information a little

We have excised the initial sentences to parse the background.

- Claiming GABA levels as a possible biomarker of motor learning: are you suggesting it as a mechanism of increased learning or predictive?

We are focused on increased learning. The predictive component of the training is intended to be derived from a bottom-up procedural process.

- Tense switching: page 4, line 9-10

We have attempted to correct tense switching.

- Unclear statement: page 4, 11-13: Recent trends in the field emphasize modifying rehabilitation approaches to increase sensitivity to individual variability to promote the reclamation of function and improve quality of life

We have attempted clarification of the text.

- Citation needed for page 4, line 14, regarding adjuvant therapies

We have added a reference to Mang et al, 2013.

- Page 4, line 23, link cerebral infarction to stroke (or call it stroke)

Thank you for that correction.

- GABA target paragraph, beginning page 4, line 20: needs more narration/explanation

We have attempted clarification of the text.

- Suggest replacing ‘advancements’ on page 5, line 14, with ‘studies’ or ‘research’ a

Corrected.

- Page 5 line 20, what is ‘practical’ learning?

Thank you for that correction.

- Unclear sentence: Identifying how GABA concentration may covary with behavior during motor learning could enable targeting this neurotransmitter system with interventions that can change GABA concentration to facilitate skill acquisition.

We have attempted clarification of the text.

- Page 6, line 9-10: GABA system's flexibility to acquire a skill – it is not the GABA system that is acquiring a skill, please reframe

Corrected.

- The motivation for your participant groups, starting page 6, line 22 is disjointed.

We have attempted clarification.

- Participants: clarify healthy control recruitment vs. chronic stroke

Corrected.

- Split up the right hand dominance/left hemisphere stroke cells in the stroke eligibility table (table 1)

Corrected.

- Table 1: definition of ‘active substance abuse’?

Corrected to “self-reported”

- Page 10, line 6: ‘similar’ or the same imaging/MRS protocol?

Corrected.

- Define ‘spin exercise’ for the third session for stroke participants – same as other exercise session?

Apologies. This was groupthink/writing.

- Unclear statement, page 12, lines 4-6: ‘We will schedule session two on the same day as session one, but it may be scheduled separately as needed based on participant availability and scheduling preferences.’

We have attempted clarification.

- Why are some assessments underlined in Table 3?

Apologies. The error has been addressed.

- Mismatch of ‘1-2-3-4’ in Figure 1 vs. ‘2-3-4-5’ in text (page 15, line 25) is confusing as a reader: suggest using 1-2-3-4 consistency. Similarly consistency of little finger vs. pinky finger.

Corrected.

- ‘Errors are denoted as incorrect button presses within the display of a target (1 second)’: where is target presented? Unclear sentence

Corrected/clarified.

- Reference: Borg 6-20 scale, page 18 and line 5

Added reference.

- Two citations when discussion the Heiss study in the discussion

Corrected.

- Use of ‘magnet’ to describe the MRI scanner is quite casual, suggest replacing it with MRI scanner or similar

Adjusted, as appropriate.

- The meaning of entrainment is not interchangeable with training, I suggest training should be used

With respect, the use of entrainment is preferred for reporting consistency in the protocol, as this is the name used for the paradigm on the computer and score sheets.

7. PLOS authors have the option to publish the peer review history of their article (what does this mean?). If published, this will include your full peer review and any attached files.

If you choose “no”, your identity will remain anonymous but your review may still be made public.

Do you want your identity to be public for this peer review? For information about this choice, including consent withdrawal, please see our Privacy Policy.

Reviewer #1: No

Reviewer #2: Yes: Mark Mikkelsen

Reviewer #3: No

[NOTE: If reviewer comments were submitted as an attachment file, they will be attached to this email and accessible via the submission site. Please log into your account, locate the manuscript record, and check for the action link "View Attachments". If this link does not appear, there are no attachment files.]

While revising your submission, please upload your figure files to the Preflight Analysis and Conversion Engine (PACE) digital diagnostic tool, https://pacev2.apexcovantage.com/. PACE helps ensure that figures meet PLOS requirements. To use PACE, you must first register as a user. Registration is free. Then, login and navigate to the UPLOAD tab, where you will find detailed instructions on how to use the tool. If you encounter any issues or have any questions when using PACE, please email PLOS at figures@plos.org. Please note that Supporting Information files do not need this step.

In compliance with data protection regulations, you may request that we remove your personal registration details at any time. (Remove my information/details). Please contact the publication office if you have any questions.
